# Integration of local and systemic immunity in ovarian cancer: Implications for immunotherapy

**DOI:** 10.3389/fimmu.2022.1018256

**Published:** 2022-11-10

**Authors:** Alicja Rajtak, Marta Ostrowska-Leśko, Klaudia Żak, Rafał Tarkowski, Jan Kotarski, Karolina Okła

**Affiliations:** ^1^ 1st Chair and Department of Oncological Gynecology and Gynecology, Medical University of Lublin, Lublin, Poland; ^2^ Chair and Department of Toxicology, Medical University of Lublin, Lublin, Poland; ^3^ 1st Chair and Department of Oncological Gynaecology and Gynaecology, Student Scientific Association, Medical University of Lublin, Lublin, Poland; ^4^ Department of Surgery, University of Michigan Rogel Cancer Center, Ann Arbor, MI, United States

**Keywords:** immune cells, ovarian cancer, heterogeneity, tumor microenvironment, immunotherapy, biomarkers, TME, multi-omics

## Abstract

Cancer is a disease that induces many local and systemic changes in immunity. The difficult nature of ovarian cancer stems from the lack of characteristic symptoms that contributes to a delayed diagnosis and treatment. Despite the enormous progress in immunotherapy, its efficacy remains limited. The heterogeneity of tumors, lack of diagnostic biomarkers, and complex immune landscape are the main challenges in the treatment of ovarian cancer. Integrative approaches that combine the tumor microenvironment – local immunity – together with periphery – systemic immunity – are urgently needed to improve the understanding of the disease and the efficacy of treatment. In fact, multiparametric analyses are poised to improve our understanding of ovarian tumor immunology. We outline an integrative approach including local and systemic immunity in ovarian cancer. Understanding the nature of both localized and systemic immune responses will be crucial to boosting the efficacy of immunotherapies in ovarian cancer patients.

## Introduction

**Figure 1:**
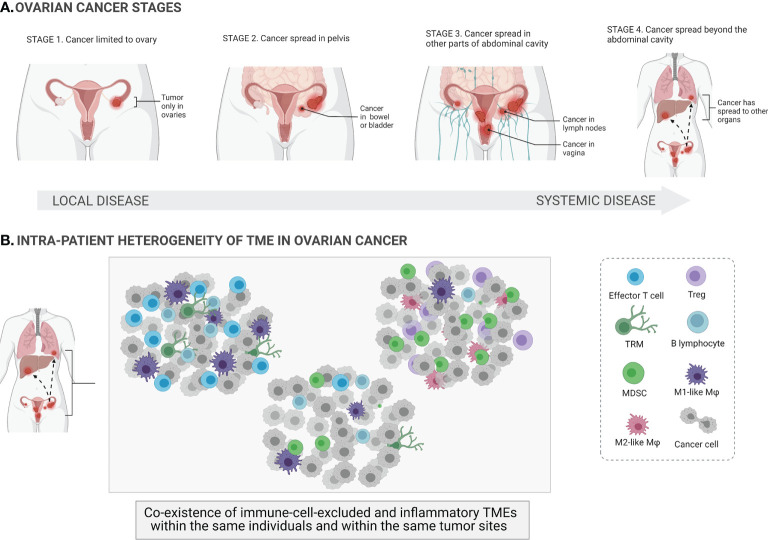
Ovarian cancer stages and heterogeneity of the tumor microenvironments. At its earliest stage (stage 1), the tumor (shown in the figure as red masses) is limited to one or both ovaries (local cancer *in situ*) and the ovarian capsule is intact. Once the capsule is disrupted, the tumor spreads beyond the confines of the ovaries (stages 2–4; systemic spread of cancer to other parts of organs) **(A)**. The tumor microenvironment (TME) of ovarian cancer involves a mixture of different immune cells, e.g., effector T cells, regulatory T cells (Tregs), tissue-resident memory T cells (TRM), B cells, macrophages (Mφ), and myeloid-derived suppressor cells (MDSCs). Ovarian cancer is a highly heterogeneous disease, and different TMEs can coexist within the same individuals and within the same tumor sites **(B)**.

Cancer is a heterogeneous disease in which the local and systemic immune responses play an important role in determining tumor growth and clinical outcomes. Over the last decade, immunotherapy revolutionized cancer treatment, yet it exhibits low efficacy in ovarian cancer (OC). OC is the deadliest among gynecological cancers in the world (1). High mortality is caused by late diagnosis, rapid disease development, relapse, and resistance to therapies (2). Unfortunately, late stages of the disease are associated with local and distant metastases, which render the disease systemic (3) (**Figure 1A**). Seventy-five percent of patients are diagnosed at advanced stages (stage III or IV); moreover, 75% of these patients die within 5 years (4). Although initial patient responses to cytoreductive debulking surgery and chemotherapy are often sufficient, most patients will develop recurrence of disease within 12–18 months after first-line chemotherapy. In contrast, among patients with early-stage disease (stage I or II), the long-term survival rate (>10 years) is 80%–95% (5).

The main challenges in treating OC include the significant heterogeneity of tumors, lack of diagnostic biomarkers, the complex tumor microenvironment (TME), and the dual role of the immune system. On the one hand, some subtypes of immune cells, e.g., dendritic cells (DCs), cytotoxic T cells, and natural killer (NK) cells, can eradicate tumor cells (immunostimulatory TME). On the other hand, other immune cells, e.g., M2-like macrophages, myeloid-derived suppressor cells (MDSCs), regulatory T cells (Tregs), can have protumor functionality and actively support tumor development (immunosuppressive TME) (6). The paradox of OC immunity is that both TMEs can coexist within the same patients and within the same tumor sites, indicating vast dynamics and variability in immune cell infiltration. Indeed, the coexistence of both immune cell-excluded and immune cell-infiltrated TMEs has been observed in the same tumor sites of the same treatment-naive patients with high-grade serous ovarian cancer (HGSOC) (**Figure 1B**). This is a major challenge for the successful application of (immuno)therapies that target the TME in OC. Furthermore, it has been shown that chemotherapy promotes local immune activation, indicating that chemotherapy can enhance the immunogenicity of immune-excluded HGSOC tumors (7).

Moreover, the TME is a dynamic niche where cellular components (immune, tumor, and stromal cells) interact with non-cellular components, i.e., secreted molecules (e.g., cytokines, growth factors, metabolites, and others). This complex network of interactions plays a key role in cell survival, invasion, and metastasis and contributes to the escape of the tumor from immune surveillance (8). Indeed, OC predominantly metastasizes along the peritoneum and distant metastatic sites including the lymph nodes, pleura, liver, and lungs. The detailed mechanisms of this metastatic cascade are largely unknown, yet evidence has shown that OC possesses metastatic tropism for the adipose-rich omentum, which has a crucial role in the maintenance of the metastatic TME in the intraperitoneal cavity (8). It is well known that circulating tumor cells (CTCs) can course through the bloodstream as single cells or as cell aggregates, i.e., CTC clusters. Interestingly, these clusters are often observed together with immune cells, which can promote the aggressiveness of the clusters and enhance the capacity to metastasize (9). As metastasis is associated with up to 90% of all cancer deaths (10), more studies on the role of systemic immunity during cancer dissemination in patients will be needed to better understand this process.

Importantly, single-cell RNA sequencing (scRNA-seq) enables deep exploration of immune cell subsets in different types of cancers and the examination of the transcriptional basis of response to therapies. Although tumor-infiltrating T lymphocytes (TILs) are mainly associated with better prognosis and response to immune checkpoint inhibitors (ICIs), scRNA-seq reveals the wide diversity within this cell population, indicating that different TIL states contribute differently to tumor control and response to (immuno)therapies (11). Antigen-specific TILs differentiate into both terminally differentiated T-cell factor 1 (TCF1)^-^ exhausted effector T (Tex) cells and self-renewing TCF1^+^ precursor exhausted T (Tpex) cells. It has been shown that Tpex cells are responsible for the long-term maintenance and generation of effector T cells in response to ICIs. Increased Tpex cell level is associated with better patient survival (12). Therefore, targeting Tpex cells can be key for successful immunotherapeutic approaches.

It is well known that effective immune responses involve a coordinated action across different cell types and tissues that create the cancer-immunity cycle. This cycle can be divided into seven major steps, starting with the release of cancer cell antigens (step 1) *via* cancer antigen presentation (step 2), priming and activation (step 3), trafficking of T cells to tumors (step 4), infiltration of T cells into tumors (step 5), recognition of cancer cells by T cells (step 6), and ending with the killing of cancer cells (step 7) (13, 14). However, most studies on tumor immunity focus on local or systemic (peripheral) immune responses, and there is a lack of simultaneous and integrative analysis of different environments, i.e., blood, ascites, and tumor tissue in a large OC patient cohort. There is increasing evidence that both local and systemic (peripheral) immune responses are needed for effective antitumor activity. It has been shown that tumor rejection requires immune cells beyond the TME to facilitate peripheral immune activation (14, 15); even for therapy delivered intratumorally, a systemic immune response was needed for tumor rejection (14). An integrative approach that combines the local tumor niche with systemic immunity is urgently needed to confront the difficulties with treating this disease. Interestingly, using liquid biopsies that analyze cell-free DNA in bodily fluids can serve as useful and noninvasive methods for the selection of targeted immunotherapies and monitoring of cancer progression (16).

In this review, we summarize current knowledge regarding local and systemic immunity in OC. We discuss the clinical relevance of local and systemic immune cells and the soluble mediators involved in disease. Finally, we outline the critical importance of both immune components to more comprehensively understand tumor immunity and to design effective immune-based therapies in OC.

## Metastasis and immune heterogeneity in ovarian cancer

More than two-thirds of patients are diagnosed at advanced stages of OC (17), in which the tumor has metastasized beyond the confines of the ovaries. One of the major sites of OC metastasis is the omentum that is composed predominantly of fatty tissue (18). The omentum is a central regulator of peritoneal homeostasis, inflammation, fluid exchange, and angiogenesis and serves as a major source of stem cells and various immune cells (19). Indeed, omental adipose tissue contains a source of immune cell aggregates so-called “milky spots”, which contain myeloid cells, B and T lymphocytes, and other immune cells (20). Importantly, omental “milky spots” are the major source of retinoic acid required for the generation of intraperitoneal macrophages that can drive the immunosuppressive TME (8). During OC metastases, the peritoneal TME in which malignant ascites (peritoneal fluid) accumulates represents an immunosuppressive milieu that includes cancer cells, different immune cell types, and numerous tumor-promoting soluble mediators (18, 21, 22). Indeed, not only cellular components of the TME but also soluble signaling factors shape the metastatic niche in the peritoneal cavity, which augments the complexity of the TME. Thus, ascitic fluid provides the opportunity to assess the components of the TME that may serve as valuable clinical biomarkers of the status of disease or to evaluate the potential effect of different therapeutic approaches to assess the antitumor immune response. However, more data are needed to evaluate whether a similar pattern of ascitic fluid components also can be found in the peripheral blood. Using peripheral blood-circulating immune biomarkers can be a valuable approach for designing simple blood tests in clinical practice.

Another major challenge that remains is the high heterogeneity of ovarian tumors that substantially impedes treatment efficacy. Analysis including whole-exome sequencing, RNA-seq, immunohistochemistry, neoepitope prediction, and *in situ* T-cell receptor (TCR) sequencing of metastatic sites of the TME revealed intersite immune heterogeneity. The progressing metastatic sites were characterized by immune cell exclusion, whereas stable and regressing metastatic sites were infiltrated by both CD4^+^ and CD8^+^ T-cell populations and showed oligoclonal expansion of T cells. On the one hand, progressing metastases were characterized by immune suppression and upregulation of Wnt signaling, higher genetic alterations in human leukocyte antigen (HLA) molecules and neoepitope loads. On the other hand, regressing metastases revealed antitumor immune activation with the enrichment of interferon (IFN)-γ, HLA, C-X-C Motif Chemokine Ligand 9 (CXCL9), and TCR signaling (23).

To conclude, single-cell analyses on the protein and RNA level can be a useful tool for analyses of the TME heterogeneity at the single-cell level, leading to a better understanding of cell function. Multiparametric examination of local (i.e., primary tumor tissues, ascites) and systemic (i.e., metastatic tumor sites, peripheral blood) immunity is critical to better understand the biology and immune responses of OC tumors and design potentially effective immunotherapies.

## Local (tumor) immunome of ovarian cancer

The tumor niche involves a mixture of different immune cells, e.g., T cells, DCs, NK cells, tumor-associated macrophages (TAMs), MDSCs, and Tregs, which are engaged in both tumor suppression and tumor progression (24). These cells produce various signaling factors such as cytokines, chemokines, growth factors, and other signaling molecules that shape the dynamic communication network and clinical outcome in OC patients (**Table 1**).

**Table 1:** Tumor tissue immune profiles and their clinical significance in ovarian cancer.

Type of immune cells/markers	Phenotype	Clinical relevance	Ref
T cells	CD8^+^ CD3^+^	↑ CD8^+^ T cells are associated with prolonged OS, DSS	(25) (26) (27) (28)
		↑ CD3^+^ and CD8^+^ T cells are associated with increased DSS	(29)
		↑ CD3^+^/CD8^+^ T cells are associated with low stage	(30)
		↑ CD8^+^ T cells are associated with improved OS	(31) (32) (33) (34) (35) (36)
		↑ CD8^+^ T cells are correlated with higher histopathological grade and advanced stage	(37)
		↑ CD8^+^ T cells are associated with better OS, PFS	(38)
	TIM3^+^ CD127^+^ CD4 ^+^γδT	↑ CD8^+^ TIM3^+^ CD127^+^ T cells are associated with better OS ↑ CD4^+^ γδT cells are associated with reduced OS	(39)
		↑ CD8^+^ is correlated with shorter DFI OS ↑ CD3^+^ is associated with clinical responsiveness to first-line chemotherapy	(40)
	CD45^+^ CD8^+^	High level of CD8^+^ cells and a high CD8^+^/Foxp3^+^ ratio are associated with increased DSS CD8^+^ CD45^+^ Foxp3^+^ cells or a high CD8^+^/Foxp3^+^ ratio is associated with an increased DSS in advanced stage High CD8^+^/Foxp3 ratio is associated with improved OS and PFS The presence of CD45^+^ and Foxp3^+^ cells in omental metastases is associated with an increased DSS	(41) (42) (28)
	CD8^+^/Treg CD8^+^/CD4^+^	High CD8^+^/Treg ratio is associated with better OS High CD8^+^/CD4^+^ ratio is associated with better OS	(43)
	CD45^+^ PD-1^+^ CD8^+^ CD3+	High level of stromal infiltrate of CD45^+^ and CD3^+^ cells in the omental lesions is associated with lymph node metastasis High CD8^+^ infiltrate in the peritoneal lesions compared to the primary tumors is observed in platinum-sensitive tumors High level of stromal PD-1+ cells in the peritoneal lesions is associated with reduced platinum sensitivity	(44)
	TILs	↑ TILs are associated with better OS	(45) (46)
	CD8^+^ CD103^+^	High CD103^+^ TILs are correlated with increased DSS ↑ CD103^+^ TILs are associated with prolonged OS	(35) (28)
		↑ CD8^+^CD103^+^ TILs are associated with good OS High CD103^+^ TILs are correlated with increased DSS	(47)
Th17		The number of Th17 cells is decreased in the advanced stages (FIGOIII/IV) vs. FIGO I	(48)
TRM	CD3^+^ CD8^+^ CD103^+^ PD-1^+^	CD103^+^ cells are associated with better DSS CD103^+^ and PD-1^+^ cells are associated with increased DSS	(49) (35)
		CD3^+^ CD103^+^ cells are associated with better DSS in patients after primary surgery and adjuvant chemotherapy ↑ CD103^+^ cells are associated with better OS	(50)
Tregs	CD4^+^ CD25^+^ Foxp3^+^	↑ Tregs are associated with reduced OS	(51)
	CD4^+^ CD25^+^ CD12^-^ Foxp3^+^	Tregs increase with disease progression	(48)
		↑ Foxp3 is associated with worse OS	(33) (52)
	CD4^+^ Foxp3^+^ Th17	↑ Treg^+^ Th17 ratio is associated with reduced OS, PFS	(53)
B cells	CD20^+^	High level associated with better OS and DSS High expression of CD20 correlated with high tumor grade	(54) (31) (29)
DCs	LAMP^+^	↑ LAMP^+^ cells are associated with longer RFS and OS	(55)
	CD1a^+^	↑ cells are associated with better survival rate	(56)
pDC	CD4^+^ CD123^+^ BDCA2^+^	↑ pDC is associated with early relapse	(57) (58)
NK	CD16^+^	↑ associated with worse OS	(59)
	CD56^+^	↑ associated with better OS	(60)
	CD57^+^	↑ CD57^+^ is associated with better OS	(27)
TAMs	CD163^+^ CD68^+^	↓ CD163/68^+^ ratio is associated with better OS and PFS	(61) (46)
	CD163^+^ COX-2^+^	↑ COX2^+^/TAMs ratio is associated with poor OS and RFS	(62)
	CD45RO^+^	↑ associated with better survival	(63)
	CD206^+^	↑ CD206^+^ is associated with poor OS	(64)
	CD163^+^ CD68^+^	High M1/M2-like TAMs ratio correlated with improved 5-year prognosis	(65)
	M1 CD14^+^ CD80^+^ M2 CD14^+^ CD163^+^	High level of M1/M2 ratio is associated with better OS, PFS, and PFI	(66)
MDSCs	Lin- CD45^+^ CD33^+^	↑ MDSC is associated with worse OS	(67)
	HLA-DR^-/low^ CD11b^+^ CD14^+^ CD15^-^ M-MDSC	↑ MDSC is associated with worse OS	(68)
	MDSC+VEGF	High VEGF levels correlated with MDSC migration and poor prognosis	(69)
Cytokines and others	IL-8	↑ associated with poor OS and PFS	(70) (71) (72)
	IL-6 IL-10 IFN-γ	↑ IL-6/IL-10 is associated with poor OS ↑ IFN-γ is associated with increased OS	(73) (74)
	IL-22	↑ associated with better OS	(75)
	CCR1	↑ associated with reduced DFS	(76)
	CCR3	↑ CCR3 associated with increased OS	(77)
	CCL18	↑ associated with reduced OS and metastasis	(78)
	CCL28	High CCL28 levels associated with recruitment of Tregs cells and poor disease outcome	(79)
	CXCL2	↑ associated with worse OS	(80)
	CXCL9 CXCL10	↑ associated with better OS	(60)
	CXCL13	↑ associated with longer OS, PFS	(81)
	CXCR5	↑ associated with prolonged survival	(81)
	CXCR6 CXCL16	↑ associated with metastasis	(82)
	CXCR3	↑ associated with a reduced PFS, OS	(83)
	CXCR4	↑ associated with reduced OS and PFS	(84) (85)
	CX3CR1	↑ associated with reduced OS and PFS after chemotherapy	(86)
	CD38	High level associated with better OS	(87)
	TGF-β	High level associated with worse OS High TGFβ1 levels associated with CD8^+^ Treg induction and poor prognosis	(88) (89)
	VEGF	High level associated with poor OS	(72)
	TNF-α	High TNF levels correlated with myeloid cells recruitment and tumor progression	(90)

TIM3, T-cell immunoglobulin and mucin domain 3; IL, interleukin; γδT, gamma/delta T cells; Foxp3, forkhead box P3; TILs, tumor-infiltrating lymphocytes; LAMP, lysosomal associated membrane protein; pDC, plasmacytoid dendritic cells; BDCA2, binding of blood dendritic cell antigen 20; COX2, cyclooxygenase 2; Lin, lineage; HLA-DR, major histocompatibility complex (MHC) II cell surface receptor; IFN-gamma, interferon-gamma; CCR, C-C chemokine receptor; CCL, C-C chemokine ligand; CXCL2, C-X-C motif chemokine ligand; CXCR5, C-X-C motif chemokine receptor; CX3CR1, C-X3-C motif chemokine receptor 1; TGFB1, transforming growth factor beta 1; TNF-α, tumor necrosis factor alpha. ↑ - high; ↓- low.

### Lymphocytes

Tumor T-cell populations, i.e., CD8^+^ TILs are associated with better clinical outcomes in OC (25–27, 29, 31–42, 47, 49, 50, 52, 91). First, the presence of CD8^+^ TILs correlates with improved overall survival (OS) (26, 27, 31–33, 35, 36, 38–40). Second, OC patients with a higher infiltration of CD8^+^ TILs had prolonged progression-free survival (PFS) (28, 31, 38, 55) and disease-specific survival (DSS) (25, 28, 29, 42, 92) compared to patients with low levels of CD8^+^ TILs. Third, the infiltration of CD103^+^ tissue-resident memory T cells (TRM) was associated with better OS (28, 50) and DSS (28, 35, 49, 50).

Next, CD4^+^ Treg infiltration can be associated with a poor clinical outcome (48, 51). Patients with advanced stage III or IV OC have higher percentages of immunosuppressive FOXP3^+^ Tregs. Tumor-infiltrating Tregs were associated with reduced survival and high mortality in OC patients (51). However, multidimensional immune profiling revealed that the combination of Cytotoxic T lymphocyte antigen 4 (CTLA-4), Lymphocyte activation gene-3 (LAG-3), and Tregs is associated with improved PFS in HGSOC (93).

Finally, B cells can represent positive or negative prognostic factors. CD20^+^ B cells from HGSOC positively correlated with DSS (29). Yet, patients with residual disease or another histological subtype demonstrate lack of any significant survival benefit with CD20^+^ B-cell infiltration (34). In an independent study, it has been shown that tumor-infiltrating CD20^+^ B cells positively correlate with OS in patients with OC (31). Due to inconsistent results, further studies on B cells in different groups of OC patients will be needed to resolve their clinical relevance in the TME. Nevertheless, recent results from a large HGSOC cohort composed of 534 patients indicated that tumor B cell-derived IgA redirects myeloid cells against extracellular oncogenic drivers, which causes tumor cell death and sensitizes tumor cells to cytolytic killing by T cells (94). In contrast, regulatory B cells (Bregs) promote the conversion of FoxP3^+^ Tregs from resting CD4^+^ T cells and support cancer metastasis. Patients with OC showed high frequencies of IL-10^+^ B cells in ascites, and their level positively correlated with Foxp3^+^CD4^+^ T cells. These cells also inhibited IFN-γ production by CD8^+^ T cells, indicating that Bregs can suppress antitumor immune responses (16). Thus, B-cell immune responses in OC may be crucial for (immuno)therapy efficacy.

### Dendritic cells

DCs are responsible for antigen presentation, making them intermediaries between the innate and adaptive systems (95, 96). Two main populations of DCs have been reported: the conventional DCs (cDCs) that activate CD8^+^ T cells *via* cross-presentation and the plasmacytoid DCs (pDCs) that may be engaged in both tumor protection and tumor suppression (97). It has been shown that HGSOC patients possessing mature LAMP^+^ DCs have better Th1 immune response and favorable OS (55). Similarly, CD1a^+^ DCs were associated with better survival in OC patients (56). Recently, six DC-related prognostic genes, i.e., *CXCL9*, *UBD*, *CXCL11*, *VSIG4*, *ALOX5AP*, and *TGFBI*, were identified to construct a risk model that could stratify OC patients into two groups with different survival outcomes. In contrast to *VSIG4*, *ALOX5AP*, and *TGFBI*, three genes, i.e., *CXCL9*, *UBD*, and *CXCL11* were associated with better outcomes (98).

Although DCs infiltrate OC, they are usually dysfunctional, have weak antigen presentation activity, and downregulate surface costimulatory molecules (97). Indeed, tolerogenic DCs inhibit antitumor immunity by producing less pro-inflammatory cytokines and more immunosuppressive cytokines. First, intratumoral tolerogenic pDCs secrete less IFN-α, tumor necrosis factor-alpha (TNF-α), Interleukin 6 (IL-6), C-C motif chemokine ligand 5 (CCL5), and macrophage inflammatory protein 1 (MIP1) in OC patients. Second, pDCs induce the secretion of IL-10 from CD4^+^ T cells, contributing to immune tolerance in these patients. Third, they produce enzymes that negatively regulate effector functions of T cells, i.e., nitric oxide synthase (NOS) and indoleamine 2,3-dioxygenase (IDO). It has been shown that there was a higher level of IDO^+^ DCs in tumor-draining LNs compared to the healthy donor LNs (99–101). Moreover, accumulation of pDCs in tumors is associated with early relapse in OC patients (57, 58).

### Natural killer cells

NK cells are the first line of defense against the development of cancer and are principal effectors in antibody-dependent cell-mediated cytotoxicity (ADCC), yet the relevance of this population in OC remains controversial. Most reports highlight low infiltration of NK cells within the ovarian tumor, and cells with suppressive activity dominate (102, 103). CD16^+^ NK cells predicted worse OS (59). In contrast, infiltration of CD56^+^ NK and CD57^+^ NK was associated with better OS in OC patients (27, 60).

### Myeloid cells

TAMs and MDSCs are the largest groups of myeloid cells in the TME (104).

TAMs can represent two major phenotypical dichotomy, i.e., antitumor M1-like macrophages and protumor M2-like macrophages (5). In OC, TAMs with M2-like phenotype predominantly exist, which drive tumor invasion, angiogenesis, metastasis, and recurrence (74, 105, 106). Indeed, in the malignant ascites of OC, abundant M2-like protumoral TAMs can be found (107). TAM/M2 macrophage frequencies were found to be positively associated with OC stage and ascites volume (107–109). In contrast, M1/M2 ratio was negatively associated with OC stages (65). Both the M1/M2 and M2/TAM ratios have been shown to be positively associated with PFS and OS in OC patients, yet overall, TAM density shows no prognostic relevance (65, 109, 110). M2 density in the ascites is associated with reduced recurrence-free survival (RFS) (74) and PFS (109, 110). It has been shown that CD163^+^Tim4^+^ resident omental macrophages are responsible for the metastatic spread of OC cells, and their genetic or pharmacological depletion inhibits tumor progression and metastatic spread (111). Similarly, using an *in vivo* xenograft OC model, it has been shown that depletion of intraperitoneal macrophages, but not neutrophils or NK cells, reduces the peritoneal metastasis and tumor progression of OC (112).

MDSCs are the key component in immunosuppressive networks (113). Three subsets of these cells exist in humans, i.e., CD33^+^HLA-DR^-/low^CD14^+^CD15^-^ M-MDSCs that share phenotypic and functional features with monocytes/macrophages, CD33^+^HLA-DR^-/low^CD14^-^CD15^+^ PMN-MDSCs that are similar to neutrophils, and CD33^+^HLA-DR^-/low^CD14^-^CD15^-^ early-stage early stage myeloid-derived suppressor cell (eMDSCs) that present more immature cell populations. MDSCs are absent (or present at a very low level) in healthy individuals, whereas they constitutively appear in elevated number in cancers, e.g., in blood, tumor tissue, bone marrow, lymph nodes, and spleen (114, 115). MDSCs were significantly increased in the peripheral blood, ascites, and tumor in OC patients (68, 116, 117). First, tumor-infiltrating CD33^+^ MDSCs were significantly associated with shorter OS and reduced disease-free interval (DFI) in HGSOC patients (67). Second, IL-6/IL-10 from ascites synergistically expands CD14^+^HLA-DR^-/low^ M-MDSCs in OC patients, and high abundance of ascites/blood-derived MDSCs was associated with a poor prognosis (118). Third, increased MDSCs significantly correlate with decreased intratumoral CD8^+^ T-cell infiltration and shorter survival (69). Our group demonstrated the existence of all three MDSC subsets in all paired samples from three different environments, i.e., peripheral blood, ascites, and tumor tissue. We observed significantly higher frequencies of M-MDSCs in all three examined environments in OC patients compared to the control group; high levels of both blood-circulating and tumor-infiltrating M-MDSCs were correlated with worse OS in OC patients (68). Thus, it indicates the importance of local and peripheral immune responses.

### Soluble factor profile

Soluble mediators released by both immune and cancer cells into the microenvironment can shape the immune response and function as biomarkers (**Table 1**). The ascites ecosystem can create an immunosuppressive and metastatic environment for OC cells. A key regulator of these processes is transforming growth factor-beta (TGF-β), which promotes survival of OC stem cells, epithelial-to-mesenchymal transition (EMT), and chemoresistance (119). It has been shown that TGF-β is elevated in the ascites of OC patients (120, 121), and blockade of TGF-β signaling limits immune exclusion and improves the chemotherapy response in metastatic OC mouse models (122).

Ovarian tumor–derived soluble factors stimulate neutrophils to create neutrophil extracellular traps (NETs) that promote the OC premetastatic niche. NETs were observed in the omentum of the mouse model of OC and of women with early-stage OC (123).

Using proteomic analysis, 779 proteins in the ascites samples of HGSOC patients have been identified as clinically relevant; CAPG, LCK, and TNFAIP6 have 91.2% correctness in identifying short-term survivors (124). Similarly, multiplex cytokine array analysis of 120 cytokines in the malignant ascites of OC patients showed that high levels of osteoprotegerin (OPG), IL-10, and leptin were associated with shorter PFS (125). However, it is unknown whether the profiles of these soluble markers in the ascites reflect their status in the blood samples.

### Multiparametric analysis of local immunome

An increasing number of studies focus on multiparametric analysis of the immune component in cancer patients.

A recent study characterized ascitic fluid using scRNA-seq to profile ~11,000 cells of 11 patients with HGSOC. Results showed significant interpatient variability in the composition of ascites cells, including dichotomous macrophage populations. One population was enriched with major histocompatibility complex (MHC) class II, IFN-γ receptor 1, and M1-associated genes and the other with complement factors, suggesting the existence of both phenotypes in the ascites. Yet, it is unknown whether similar dichotomous macrophage subpopulations exist in the paired tumor and blood samples (126). Moreover, a recent study estimated 22 immune cell subsets from databases with more than 2,000 HGSOC patients who underwent platinum-based chemotherapy. Results showed that a high level of M1 and M0 in tumor tissue was associated with better OS. Neutrophils were associated with poor OS. Among the immunoreactive tumors, the M0 macrophages and the CD8^+^ T cells were associated with improved OS, whereas the M2 macrophages showed worse OS; programmed death receptor-1 (PD-1) was associated with good OS and PFS in this subtype (127).

Furthermore, three different immune types (A, B, and C) have been identified using the expression of immune‐related genes of 307 OC samples. Patients in subtype B had poorer prognosis and lower survival rate. Moreover, the predictive response rate to immunotherapy in type B was significantly higher than that in types A and C; patients in immune type B have a superior response to immunotherapy. Immune subtype B was characterized by low levels of M1 macrophages and Th cells and high levels of Treg‐type macrophages and M2 macrophages. IL‐6‐Janus kinase - signal transducer and activator of transcription 3 (JAK-STAT3) pathway activity was increased in the immune subtype B. In contrast, enrichment of KRAS‐downregulation pathway increased in both A and C immune types with superior prognosis (128).

It is well known that different patterns of T-cell accumulation in the tumor niche, i.e., immune infiltrated (a), excluded (b), and desert (c), shape different responses to immunotherapies. scRNA-seq analysis of 15 ovarian tumors showed that predysfunctional CD8^+^ GZMK T cells are enriched in the excluded tumors, while FCN1 monocytes and immature MARCO macrophages are enriched in desert tumors (129). Yet, it is unknown whether the profiles of immune cells in the tumor niche reflect their status in the ascites or peripheral blood.

Interestingly, recent data of tumor-immune niche single cells, derived from 44 tumors, showed that HGSOC patients with *BRCA1/2* gene mutations had better immune response against tumors and distinct immune cell landscape compared to patients without mutations (130). Thus, different (immuno)therapeutic strategies for these clinical subgroups may be needed.

Using transcriptomic analysis of OC, three immunogenomic subgroups have been proposed, i.e., hyperimmunogenic (a), moderately immunogenic (b), and hypoimmunogenic (c). Activated DCs, M1 macrophages, CD8^+^ T cells, follicular helper T cells, and CD4^+^ memory T cells were enriched in the hyperimmunogenic subtype. Intriguingly, this subgroup had the highest expression of *PD-L1* (*programmed death ligand-1*), *PD-1*, and *PD-L2*. Clinically, the hyperimmunogenic subtype had an early International Federation of Gynecology and Obstetrics (FIGO) stage and better survival prognosis and response to immunotherapy compared to those of the moderately immunogenic and hypoimmunogenic subtypes (131).

Finally, three different immunometabolism subtypes of OC were identified, i.e., “immune suppressive-glycan metabolism subtype” with high levels of immunosuppressive cell infiltration and glycan metabolism activation (a), “immune inflamed-amino acid metabolism subtype” with abundant adaptive immune cell infiltration and amino acid metabolism activation (b), and “immune desert-endocrine subtype” with low immune cell infiltration and upregulation of hormone biosynthesis (c). Results showed that “immune inflamed-amino acid metabolism subtype” was more sensitive to chemotherapy and displayed a significantly better response to immunotherapy compared to “immune suppressive-glycan metabolism subtype” and “immune desert-endocrine subtype” (132). Therefore, immunometabolism subtypes may have a predictive value for (immuno)therapy stratification.

In the future, integration of multiparametric analysis including single-cell analysis on transcriptomic, proteomic, and metabolomic level is needed to understand the heterogeneity of OC and to boost (immuno) therapy efficacy.

## Peripheral immunome of ovarian cancer

The tumor niche can influence the systemic immune macroenvironment status, thereby making opportunities for simple and noninvasive blood biomarkers for patient immunostratification and design of immunotherapy. The development of predictive blood-based immune biomarkers for cancer monitoring is of interest; yet, until now, a peripheral immune biomarker that can be used in bedside decision-making in oncology is lacking (15). Nevertheless, human studies demonstrate an association between peripheral immunome and clinical outcome of OC patients (**Table 2**).

**Table 2:** Circulatory immune profiles and their clinical significance in ovarian cancer.

Type of immune cells/markers	Phenotype	Clinical relevance	Ref
T cells	CD45^+^ CD3^+^	↓ T cells are associated with reduced OS	(133)
Th22	CD4^+^ IFNγ^-^ IL17^-^ IL22^+^	↑ Th22 cells are associated with higher tumor stage	(134)
Th17	CD4^+^ IL17^+^ IFNγ^-^	↑ Th17 cells are associated with higher tumor stage	(135)
Tregs	CD4^+^ CD25^+^ Foxp3^+^	High level of Tregs is a significant predictor of OC early relapse	(136)
B cells	CD45^+^ CD3^-^ CD16^-^ CD56^-^ CD19^+^	↓ B cells are associated with reduced OS	(133)
DCs	CD4^+^ CD123^+^ BDCA2^+^	High density of pDC correlated with poor disease outcome	(57)
NK	CD3^-^ CD16^+^ CD56^+^	↓ NK is associated with poor OS	(137)
TAMs	CD14^+^ CD80^+^ Glut^+^ CD14^+^ CD163^+^	↑ M1/M2 is associated with higher OS, PFS	(66)
MDSCs	HLA-DR^-^ CD14^+^	↑ MDSC is associated with shorter RFS	(118)
	HLA-DR^-/low^ CD11b^+^ CD14^+^ CD15^-^	↑ M-MDSC is associated with worse OS	(68)
	CD3^-^ CD19^-^ CD56^-^ HLA-DR^-/low^ CD14^+^ CD15^-^	↑ M-MDSC is associated with decreased survival	(116)
Chemokines/cytokines	IL-6 IL-8	↑ IL6 and IL-8 are associated with reduced OS, DFS ↑ IL-8 is associated with poor OS and PFS	(138) (139) (70)
	CXCL1 CXCL2	↑ associated with reduced OS	(140)
	CCR3	↑ associated with increased OS	(77)
	CCL4 CXCL1 CCL20	↑ associated with shorter RFS, OS	(141)
	CCL22	High CCL22 levels correlated with recruitment of Tregs and poor disease outcome	(51)

DCs, dendritic cells; DFI, disease-free interval; DFS, disease-free survival; DSS, disease-specific survival; MDSCs, myeloid-derived suppressor cells; NK, natural killer; OS, overall survival; PD-L1/PD-1, programmed death ligand/receptor-1; PFS, progression-free survival; TAMs, tumor-associated macrophages; Tregs, regulatory T cells; TRM, tissue-resident memory T cells; VEGF, vascular endothelial growth factor; RFS, relapse-free survival. ↑- high; ↓- low.

The gold standard markers for monitoring OC patients are Cancer Antigen 125 (CA 125) and Human epididymis protein 4 (HE4). However, their specificity is low. First, CA-125 sensitivity is only 50% in stage I OC (142). Second, a higher level of CA-125 has been reported during menstruation, early pregnancy, endometriosis (143), and peritoneum inflammatory diseases (144). Third, HE4 is better than CA125 in diagnosing patients with OC due to higher specificity, yet HE4 increases with age, smoking, and renal diseases (145).

Recent studies have proposed an analysis of the serum-functional immunodynamic status (sFIS) in OC patients. The concept of this “*in sitro*” (*in vitro* plus *in situ*) assay implies using human myeloid cells that are exposed to patients’ serum (*in vitro*) to assess serum-induced (si)-Nuclear Factor Kappa B (NFκB) or IFN/interferon-stimulated gene (ISG) responses (as active signaling reporter activity) within them, thereby mimicking patients’ *in situ* immunodynamic status. First, the assay can decode peripheral immunity (by indicating higher enrichment of si-NFκB over si-IFN/ISG responses). Second, it estimates survival trends (si-NFκB or si-IFN/ISG responses associated with negative or positive prognosis, respectively). Third, it coestimates the malignancy risk (relative to benign/borderline ovarian lesions). Data revealed the abundance of protumoral myeloid si-NFκB response^HIGH^si-IFN/ISG response^LOW^ inflammation in periphery of patients with OC. Interestingly, in the mouse metastatic OC model, the sFIS assay predicted the higher capacity of chemoimmunotherapy (paclitaxel–carboplatin plus anti-TNF antibody combination) in achieving a proimmunogenic peripheral status (si-IFN/ISG response^HIGH^si-NFκB response^LOW^), which is aligned with a high antitumor efficacy (146). Thus, the sFIS assay can be beneficial in personalized patient monitoring, immunostratification, and (immuno)therapeutic decision-making in OC.

Moreover, the association of three inflammation-based parameters with the survival of OC patients has been proposed, i.e., lymphocyte/monocyte ratio (LMR) (a), neutrophil/lymphocyte ratio (NLR) (b), and platelet/lymphocyte ratio (PLR) (c). High NLR and PLR and low LMR were independent prediction factors of poor OS and PFS in OC (147).

Recently, it has been proposed that the blood M-MDSCs/DCs ratio is an independent predictive factor for OC survival (116). Furthermore, our study showed a positive correlation of sPD-L1 with PD-L1^+^ M-MDSCs/macrophages in the blood of pretreatment OC patients, yet no prognostic relevance was demonstrated (148). As the efficacy of PD-L1 inhibitors in OC is disappointing, new checkpoint inhibitors or/and precise selection of an appropriate group of patients may be crucial to boost the effectiveness of checkpoint inhibitors.

In many studies, the analysis of blood cytokines in OC patients was either performed individually or combined with just two or three cytokines after individual assessment. As systemic cytokinome networks are complicated in OC patients, an evaluation of the pattern of soluble mediators rather than single individual cytokines can be more informative. A recent study indicated that 12 of 27 serum cytokines correlated with OC histotypes. Two OC histotypes, i.e., HGSOC and clear cell carcinoma (CCC) shared similar cytokinome signatures involved in the “hemotaxis and angiogenesis” and “Th2-type immunity”. These results indicate that HGSOC and CCC may share a systemic immunological profile (149).

A better understanding of the network of blood soluble mediators and immune cells might reveal systemic immune characteristics of OC patients.

## Immunome in therapy design

A conventional therapeutic strategy in OC is debulking surgery followed by adjuvant platinum and taxane-based chemotherapy that shapes the global immunological landscape (150). It is known that surgery induces an immunosuppressive state to support wound healing and postoperative pain. In OC patients, debulking surgery decreases Tregs in the blood on day 1 postoperatively, with an increase on day 7 postoperatively. Moreover, increased levels of TGF-β also have been observed. In contrast, chemotherapy reduces immunosuppression and promotes immunostimulation in OC patients (151). Understanding these systemic immune consequences is important for designing strategies that augment rather than impede antitumor immune responses, which can include optimal timing, dosing, or combinations.

**Figure 2:**
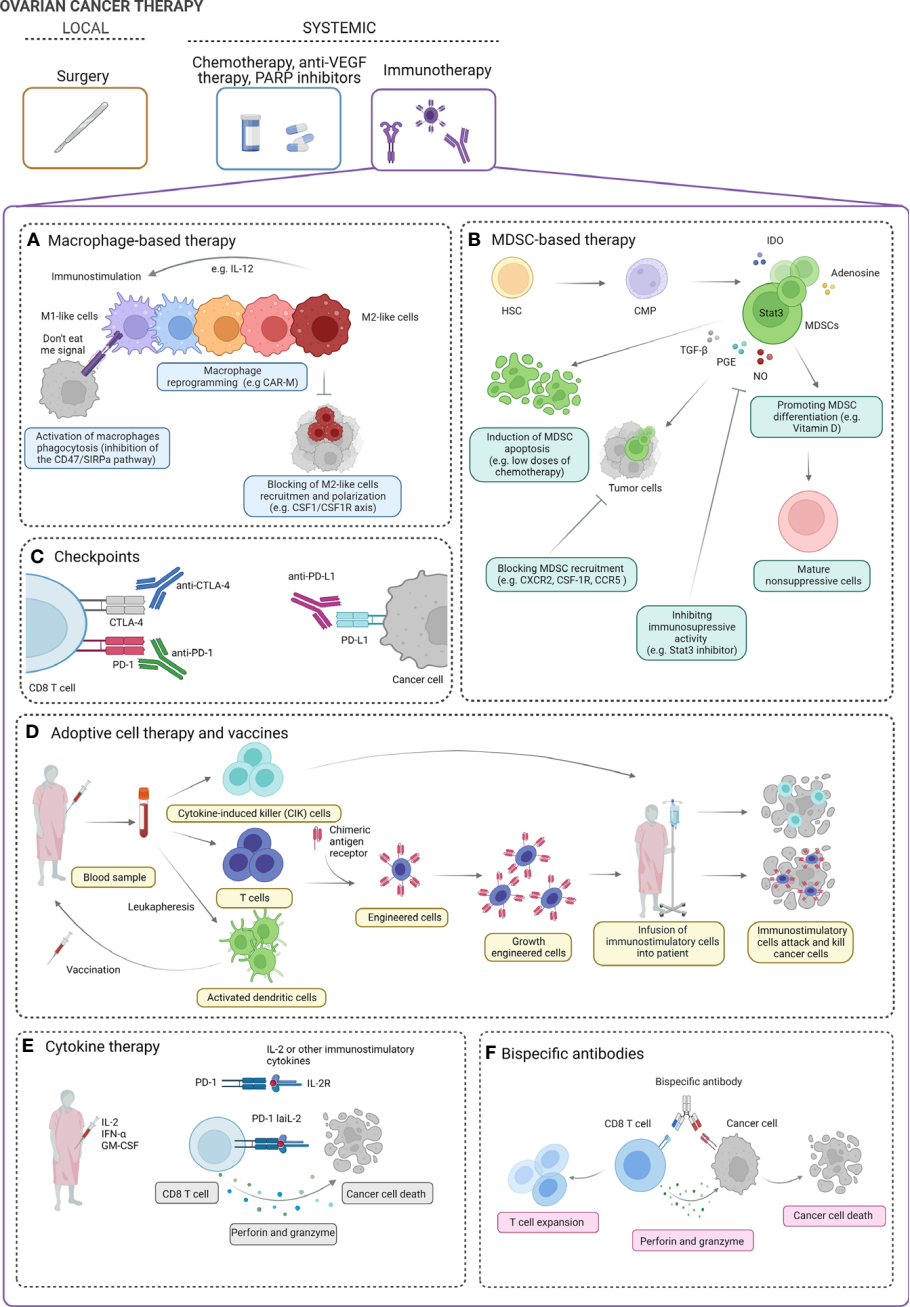
Ovarian cancer therapy. The standard treatment for ovarian cancer (OC) includes local intervention (debulking surgery) followed by systemic treatment [chemotherapy, anti-vascular endothelial growth factor (VEGF) therapy, PARP inhibitors]. Several strategies of systemic immunotherapy can be of clinical benefit in OC patients. Macrophage-based strategies can include activation of macrophage phagocytosis, macrophage reprogramming into immunostimulatory M1-like phenotype, and blocking of M2-like cell recruitment **(A)**). Myeloid-derived suppressor cell (MDSC)-based strategies include induction of MDSC apoptosis, blocking MDSC recruitment, inhibiting the immunosuppressive activity of MDSCs, and promoting the differentiation of MDSCs into mature non-suppressive cells **(B)**. Blocking of immunoinhibitory checkpoints (e.g., PD-1, PD-L1, CTLA-4, and others) can boost the immune response and promote ovarian tumor cell killing **(C)**. Cytokine-induced killer (CIK) cells, engineered immune cells (e.g., T cells), and dendritic cell vaccines can be used to boost the antitumor immune response cell activity and enhance cancer cell killing **(D)**. In clinical studies, using IL-2, IFN-α, and GM-CSF has been proposed in OC treatment. In preclinical studies, modified low-affinity IL-2 fusion protein in combination with anti-PD-1 (PD-1-laIL-2) decreases affinity for Tregs and increases avidity to CD8 TILs, which promotes better tumor control and less toxicity than single or combination treatments **(E)**. Bispecific antibodies have affinity for both the tumor-associated antigen and the CD8 effector T cells. In the presence of perforin and granzyme, they effectively target T lymphocytes to elicit antitumor effects **(F)**. IL, Interleukin; M1 and M2, macrophages; CAR-M, chimeric antigen receptor macrophage; CSF-1: macrophage-colony stimulating factor; CSF-1R, macrophage colony stimulating factor signaling through its receptor, SIRPA, signal regulatory protein alpha; HSC, hematopoietic stem cells; CMP, common myeloid progenitor; IDO, indoleamine 2,3-dioxygenase; STAT3, signal transducer and activator of transcription 3; TGF-b, transforming growth factor- beta; PGE, prostaglandin E; NO, nitric oxide; CXCR2, chemokine C-X-C motif receptor 2; CCR5, C-C motif chemokine receptor 5; CTLA-4, cytotoxic T-lymphocyte associated protein 4; PD-1, programmed cell death protein 1; PDL-1, programmed cell death ligand 1; INF-alfa, interferon alpha; GM-CSM, granulocyte macrophage colony-stimulating factor.

In recent years, we have witnessed an “immunotherapy tsunami”; however, the results of treatment based on immunotherapy are still unsatisfactory in OC (152). To overcome cancer-related immune dysfunction of cancer, an effective immunotherapy drives peripheral immune response, boosting local and systemic immunity. Multiple strategies have been proposed to modulate the immunome to enhance OC (immuno)therapy efficacy (**Figure 2**).

### Macrophage-targeted strategies

Briefly, macrophage-targeted therapies can be divided into two main strategies, i.e., limiting tumor-promoting M2-like macrophages (a) and activating tumor-suppressing M1-like macrophages (b) (153).

First, several preclinical and clinical trials exploring the restoration of phagocytosis in macrophages using the inhibition of the CD47/signal regulatory protein alpha (SIRPa) pathway have been proposed (154). CD47 acts as a “do not eat me” signal that allows tumor immune evasion (155). CD47 is overexpressed in OC patients and is associated with shorter PFS (156). Thus, CD47/SIRPa signaling pathway can be an attractive target for OC therapy.

Second, it may be of interest to use modified chimeric antigen receptor (CAR)-macrophages (CAR-M) to enhance its phagocytic activity and antigen presentation against tumor cells (157). Two drugs are being tested in clinical trials in OC, i.e., CT-0508, which treats tumor patients with relapsed/refractory human epidermal growth factor receptor 2 (HER) overexpression with anti-HER2 CAR-M (a), and MCY-M11, which uses mRNA-targeted Peripheral blood mononuclear cells (PBMCs) (including CAR-M) to express mesothelin-CAR (b) (158). TAMs are the main population of immune cells in the OC, thus using CAR-M, which can reduce the ratio of TAMs and convert M2-like macrophages to M1-like, can be of great benefit in OC treatment.

Third, the Macrophage-colony stimulating factor (CSF-1)/Macrophage-colony stimulating factor signaling through its receptor (CSF-1R) axis is the major regulator of macrophage migration and differentiation. Preclinical studies using CSF-1R inhibitor (GW2580) showed reduced tumor volume, ascites, and infiltration of M2-like macrophages in OC mouse models (159). CSF1R inhibition within a triple combination with chemotherapy and antiangiogenic treatment in platinum-resistant OC patients (66, 160).

Finally, supporting M1-like functional activity can be of clinical benefit. IFN-γ, LPS, GM-CSF, and IL-12 polarize macrophages into M1-like cells (95, 161). Interestingly, IL-12 can promote a Th1 response that polarizes macrophages into M1 phenotype. In OC, IL-12 caused reduced tumor growth and even regression. GEN-1 (gene-based IL-12 immunotherapy) has been tested in a few clinical studies (phases I–II) in OC patients (162).

### Myeloid-derived suppressor cells-targeted strategies

Our group and others already demonstrated the clinical relevance of MDSCs in OC patients (68, 69, 117, 163). Thus, targeting these cells can be of clinical significance.

A few strategies to target MDSCs have been proposed in cancer patients, e.g., induction of MDSC apoptosis, blocking of MDSC recruitment, inhibition of MDSC immunosuppressive activity, and promotion of the differentiation of MDSCs into mature non-suppressive cells (164).

In mouse studies, the anti-granulocytes (Gr)-1 antibody has been proposed to eliminate MDSCs from the TME. Unfortunately, due to the lack of a Gr-1 homolog in humans, such approach cannot be used in the human clinical setting, and there is an absence of specific inhibitors of human MDSCs. However, it is noteworthy that treatment of OC patients with gemcitabine decreases immunosuppressive MDSCs and increases M1-like macrophages (165).

The efficacy of MDSC-targeting strategies against OC is currently being studied preclinically (164). A better understanding of human MDSC biology is urgently needed to reveal how to selectively target these cells in cancer patients.

### Immune checkpoint inhibitors

Blockade of checkpoint inhibitors, i.e., PD-1 and CTLA-4, may rejuvenate the immune system and become increasingly popular in cancer treatment.

Recently, a meta-analysis including 15 clinical trials involving 945 patients was performed to assess the efficacy of anti-PD-1/PD-L1 therapy in OC. The pooled results showed that the overall response rate (ORR) was 19%. Single PD-1/PD-L1 inhibitors showed limited efficacy (ORR was 9%), while combination with chemotherapy showed better efficacy (ORR was 36%). PD-1/PD-L1 inhibitors had a higher ORR in platinum-sensitive OC than in platinum-resistant OC (31% vs. 19%) (166). Similarly, a recent summary of 20 studies where 16 clinical trials targeted PD-1 (nivolumab, pembrolizumab), PD-L1 (avelumab, aterolizumab, durvalumab), and CTLA-4 (ipilimumab, tremelimumab) reported lack of improvement in survival in OC patients, and some trials were terminated early due to toxicity or lack of response (167). In contrast, combining therapy [ICIs with chemotherapy, anti-vascular endothelial growth factor (VEGF) therapy, or Poly(ADP-Ribose) Polymerase (PARP) inhibitors] improved response rates and survival in OC patients, yet it is more toxic (167).

Intrinsic resistance to Immune checkpoint blockade (ICB) remains a challenge. Adoptive transfer of senescence-associated secretory phenotype (SASP)-boosted cells sensitizes OC to anti-PD-1. In the mouse OC model, a reduction of tumor weight and better immune response, including infiltration of DCs and activated CD8^+^CD69^+^ T cells, have been observed (168). Mechanistically, deep genomic and immune profiling of OC tumors may reveal potential targets that are responsible for the resistance to ICB and lead to the design of more effective clinical trials (167). Clinically, the improved efficacy of anti-PD-1/PD-L1/CTLA-4 therapy would require better patient selection and novel combinations of drugs (169). Interestingly, a high expression of another immune checkpoint, B7-H4, was observed in gynecologic cancers. B7-H4 expression levels inversely correlate with survival in OC patients, making B7-H4 an attractive therapeutic target (170). Finally, non-immune cells, e.g., cancer-associated fibroblasts (CAFs), promote progression and resistance to therapy in OC. Importantly, CAFs shape the immunosuppressive TME milieu and attenuate the efficacy of ICB therapy (171). Therefore, targeting CAFs may be an effective strategy to sensitize OC tumors to ICB therapy.

### Adoptive cell therapy and vaccines

In general, adoptive cell therapy (ACT) assumes using autologous or allogeneic antitumor immune cells against cancer.

The effectiveness of cytokine-induced killer (CIK) cell therapy was examined in a group of 646 OC patients after first-line treatment. CIK cells are heterogeneous immunostimulatory host effector cells, including CD3^+^ CD56^+^ NKT-like cells (a), CD3-CD56^+^ NK cells (b), and CD3^+^CD56^-^ antitumor T cells (c). CIK cells proliferate rapidly and can be obtained quickly from cancer patients *via in vitro* culture (a), exhibit strong antitumor activity (b), and possess minimal toxicity (c). The OS rates at 1, 3, and 5 years were respectively 87%, 63%, and 47% for OC patients who received CIK immunotherapy combined with chemotherapy and 65%, 44%, and 31% for control group patients who received chemotherapy alone. Patients with OC who received combined therapy exhibited prolonged OS and better PFS compared to patients with chemotherapy alone (172).

Another approach can be the use of CAR or a tumor antigen-specific TCR. Targets for CAR-T include MUC16 (mucin 16)/Ca 125, mesothelin, and folate receptor-α76–78. Targets for TCR are MAGE-A4 (melanoma-associated antigen 4), WT1 (Wilms’ tumor protein 1), and NY-ESO-1 (New York esophageal-1) (173). However, CAR T-cell exhaustion due to persistent antigen stimulation and an immunosuppressive TME is a major limitation to their efficacy in solid tumors (174). Indeed, the immunosuppressive capacity of malignant ascites in OC patients demonstrates its negative effect on adoptively transferred CAR T cells. However, CAR T cells modified to constitutively secrete IL-12 are able to overcome immunosuppression of the TME in a model of ovarian peritoneal carcinomatosis, ultimately improving antitumor activity, and are currently under study in a phase I clinical trial in HGSOC (174, 175).

Moreover, DC vaccination to induce Th17 has been proposed. The development of Th1, Th17, and folate receptor (FR)-α antibodies was observed in most OC patients. Of 18 patients, seven (39%) were recurrence-free with a median follow-up of 49.2 months (176).

Finally, the loss of HLA function is an important escape mechanism for tumors from immunotherapy. Interestingly, large-scale profiling of the immunopeptidome of OC and assessing the HLA-presented antigens can be valuable in designing a new immunotherapy (177). Indeed, HLA ligandomics identified histone deacetylase (HDAC) 1 as an important tumor antigen in HGSOC, indicating HDAC1 as a valuable target for designing new peptide vaccination in OC patients (178).

### Cytokine therapy

#### Cytokines make a bridge between local and peripheral immune responses

IL-2/4/7/12/18, IFN-α/γ, TNF-α, and granulocyte-macrophage colony-stimulating factor (GM-CSF) have been studied in preclinical cancer models, and their antitumor functions have been proposed (179). Although cytokines are easy to administer, their toxicity and lack of specificity may be a limitation for their use in clinical practice.

In a phase II trial, intraperitoneal (IP) IL-2 was administered to OC patients with platinum-resistant or -refractory disease (180). Twenty-five percent of the patients experienced a treatment response with a median survival rate of 2.1 years (181). To avoid lack of selectivity and toxicity, the solution can be delivered as an engineered fusion protein, i.e., a low-affinity IL-2 paired with anti-PD-1 (PD-1–laIL-2). Such conjugate reduced the binding of both IL-2Rα and IL-2Rβ, had lower binding to Tregs, and enhanced avidity to CD8^+^ TILs, which promoted better tumor control in mice and lower toxicity than single or combination treatments (182). Using IL-2 partial agonists that promote long-lived functional CD8^+^ T cells can be of interest in designing future clinical trials in OC patients (183, 184).

In a phase II trial, IP IFN-α alternating with cisplatin was administered to 14 OC patients with minimal residual disease as salvage treatment. Fifty percent experienced complete remissions and remained disease-free over a median follow-up of 22 months (185). Moreover, in a phase I/II trial, IP IFN-α together with carboplatin showed a response of 42.8% in OC patients who had previously received intravenous cisplatin-based chemotherapy for recurrent or refractory disease (186).

GM-CSF was evaluated in combination with recombinant IFN-γ 1b (rIFN-γ1b) in a phase II trial of patients with recurrent platinum-sensitive ovarian, fallopian tube, and primary peritoneal cancer. In the group of 59 women, the combination of GM-CSF and rIFN-γ1b with carboplatin showed a response rate of 56% (187).

### Bispecific antibodies

Innovative immunotherapeutic strategy can use bispecific antibodies (BsAb)/fusion proteins that interact with tumor antigens on cancer cells and activate receptors on immune cells. It has been shown that BsAb REGN4018 binding both MUC16 and CD3 inhibits the growth of intraperitoneal tumors in a mouse model of ovarian tumors. The efficacy was shown in both monotherapy and combination of PD-1 and VEGF inhibition (188, 189).

Similarly, BsAb mPEG × HER2 that can easily provide HER2^+^ tumor tropism to mPEGylated liposomal doxorubicin (PLD) and increase the drug accumulation in cancer cells *via* receptor-mediated endocytosis showed better cytotoxicity and therapeutic efficacy in HER2^+^ ovarian tumors as compared to non-targeted PLD (190).

So far, BsAb has been approved for the treatment of hematologic malignancies; yet, no BsAb has been approved in OC. However, a few designed BsAb drugs for solid tumors are now undergoing evaluation in phase I/II clinical trials in OC patients, e.g., EpCAM/CD3 (catumaxomab) and delta-like ligand 4/VEGF navicixizumab (OMP-305B83) (191).

## Perspectives

The local antitumor immune response cannot exist without coordinated communication with the periphery (15). Therefore, understanding immune responses to cancer should encompass global analysis across the peripheral and local immune system.

First, despite the development of high-throughput single-cell technologies, there are no studies that analyze global OC immunome in a large patient cohort both at the local level (in the tumor microenvironment, ascites) and at the systemic level (in peripheral blood, metastatic tumor sites, etc.). Yet, global immune response changes during tumor development and in response to (immuno)therapy play an important role. Pairing single-cell analyses from the different tumor sites, ascites, and peripheral blood can help the discovery of valuable biomarkers that may be easily analyzed, e.g., in the blood samples, and provide useful information to help stratification of OC patients according to their immune status and management of treatment decision. For example, it would be interesting to study the cancer-immunity cycle for individual OC patients, which allows the matching of specific immunotherapies or combinations of immunotherapies.

Second, since metastases are mainly responsible for cancer-related deaths (10), the future study of mechanistic insight on how tumor cells circulate throughout the body will be crucial. It has been proposed that some immune cells, e.g., neutrophils, support CTCs leading to enhanced metastasis formation (9). However, the role of immunity in the metastatic spread of OC can be even more complex, as recent evidence suggests that CTC release relates to circadian rhythm. Intriguingly, a study shows that more than 78% of all the CTCs obtained were from the human breast cancer samples taken during the resting (sleep) phase (192). The time-dependent nature of CTCs and hence components of the immune system should be considered in future studies on the OC immunity. From the clinical point of view, time-controlled treatment might be needed to achieve maximally effective therapy.

Third, it would also be valuable to explore which anatomic sites drive antitumor immunity and which parameters/immune cells (in peripheral blood) may provide a means for noninvasive monitoring during (immuno)therapy and discovery of new biomarkers. Using cancer liquid biopsies can open new vistas of future work in this field.

Finally, it is worthy to highlight the importance of encouraging and supporting holistic basic research on the global immunome in OC patients, which can help increase the effectiveness of clinical trials.

Overall, global and integrative analysis of both local and systemic immune responses in OC can help understand tumor control and finally increase the effectiveness of (immuno)therapy.

## Author contributions

Author contributions: conceptualization, writing, review, editing: AR, MO-L, KŻ, RT, JK, KO. All authors contributed to the article and approved the submitted version.

## Funding

This work was supported in part by research grants from the National Science Centre (Opus 19 2020/37/B/NZ5/01984 to JK, and Preludium 16 2018/31/N/NZ2/02338 to KO).

## Acknowledgments

We thank M. Bobiński and T. Maj for scientific input. We thank M. Pitter for the editorial assistance.

## Conflict of interest

The authors declare that the research was conducted in the absence of any commercial or financial relationships that could be construed as a potential conflict of interest.

## Publisher’s note

All claims expressed in this article are solely those of the authors and do not necessarily represent those of their affiliated organizations, or those of the publisher, the editors and the reviewers. Any product that may be evaluated in this article, or claim that may be made by its manufacturer, is not guaranteed or endorsed by the publisher.
